# Oral Niacin and Topical Nitroglycerin to Prevent Nicotine-Related Complications Following Facelift Surgery

**DOI:** 10.7759/cureus.88389

**Published:** 2025-07-20

**Authors:** Mobeen Shirazi, Hisham A Shirazi

**Affiliations:** 1 Otolaryngology - Head and Neck Surgery, Affiliated Ear Nose and Throat Physicians, Woodstock, USA; 2 Kinesiology, University of Michigan, Ann Arbor, USA

**Keywords:** facelift flap ischemia, niacin, rhytidectomy complications, smoking, topical nitroglycerin

## Abstract

Objective

To prove through our experience that the prophylactic use of oral niacin and topical nitroglycerin results in a reduction in nicotine-related skin flap complications following facelift surgery.

Methods

This is a retrospective, observational study. The study population consisted of 40 surgical patients (27 patients who used nicotine products and 13 non-nicotine users with signs of flap ischemia) seen over 18 months. Follow-up ranged from six months to 1.5 years. The main outcome measure was the incidence of flap ischemia in nicotine users who were placed on oral niacin and topical nitroglycerin. In addition, the time needed to improve flap ischemia was measured with the use of this therapy.

Results

There was no incidence of flap ischemia in 22 nicotine users placed on oral niacin and topical nitroglycerin. Four smokers who did have early signs of flap ischemia readily improved with the above therapy within 3-5 days. One smoker had skin slough but the wound re-epithelialized in 18 days. All 13 non-nicotine users with signs of flap ischemia, who were started on niacin and topical nitroglycerin, had an improvement in the appearance of their flap in 3-7 days (mean = 5 days). No long-term complications were recorded with the use of this therapy.

Conclusion

In patients undergoing facelift surgery, the use of oral niacin and topical nitroglycerin is a safe and effective therapy to both prevent skin flap ischemia in nicotine users and to prevent skin slough in those with an ischemic facelift flap.

## Introduction

Rhytidectomy is one of the more commonly performed cosmetic surgical procedures. Complications following rhytidectomy can have devastating consequences, especially due to the elective nature of the procedure. Nicotine use is considered a relative contraindication to facelift surgery [1]. The association between nicotine use, poor wound healing, and skin flap necrosis is well known [2]. Nicotine potentiates vasoconstriction, which decreases nutritional blood flow to the skin and reduces oxygen delivery to tissues, resulting in tissue ischemia and unsatisfactory healing after facelift surgery [1]. Following rhytidectomy, smokers have been reported to be 12.5 times more likely to develop skin necrosis than nonsmokers [3].

Niacin (vitamin B3) is a water-soluble vitamin commonly used to treat hyperlipidemia [4]. A useful side effect of niacin is its prostaglandin-mediated vasodilation, which is characterized by sudden warmth, tingling, and facial flushing [5,6]. Topical nitroglycerin (NTG) has a known benefit in the salvage of ischemic skin flaps [7]. NTG works by relaxing smooth muscle, producing a vasodilatory effect on peripheral veins and arteries, with a more prominent effect on the veins [8]. An additional benefit of topical drug delivery is the ability to bypass flap hypoperfusion due to ischemia.

Our experience shows that the perioperative use of oral niacin and early application of topical NTG for an ischemic facelift flap can help prevent flap-related complications in nicotine users. Additionally, this therapy may help salvage ischemic skin flaps in non-nicotine users as well.

## Materials and methods

Methods

A retrospective, observational study was performed using 40 patients, all of whom underwent rhytidectomy over 18 months. Patients included in the study were those who used nicotine products and/or had signs of flap ischemia in the postoperative period. Data were obtained from the clinical notes, operative reports, and photo documentation of all 40 patients. Follow-up ranged from six months to 1.5 years. Among the 40 surgical patients, 27 used nicotine products (cigarettes, nicotine substitutes), and 13 non-nicotine users with signs of flap ischemia were included. 

All patients using nicotine products were started on extended release (ER) niacin (500 milligrams (mg) daily) one week prior to surgery and began an immediate release (IR) niacin formulation on postoperative days 1-7. The patients were instructed to take the amount of IR niacin that caused a subjective facial flush and to repeat this dosage 4 times daily as detailed below. 

Instructions for the use of niacin

Individual dosing requirements needed to produce the desired “flushing” effect vary greatly from person to person. The following process is designed to help determine the correct dose. 

1. Purchase the 100 mg form of niacin. Make sure that you do not purchase the “non-flush” form of niacin since the “flushing” effect is what we are hoping to produce. 

2. Start by taking a single 100 mg tablet. If no “flushing” occurs after 30 minutes from this initial dose, then take a second 100 mg tablet. 

3. If no “flushing” occurs after 30 minutes from this second dose, then take a third 100 mg tablet. 

4. Continue this process every 30 minutes until you start feeling “flushed”. 

**Figure 1:**
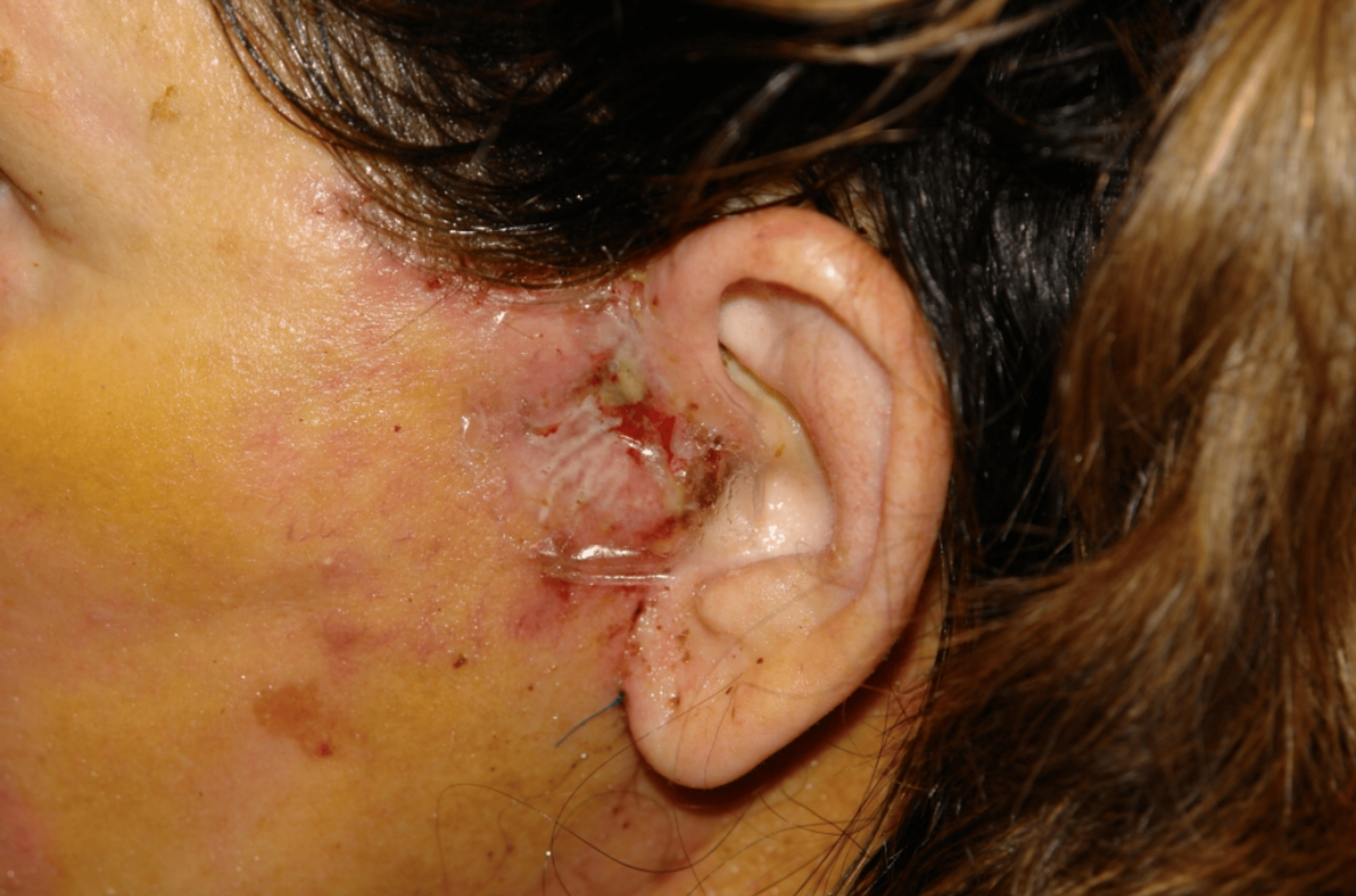
Topical application of 2% nitroglycerin (NTG) ointment in an ischemic area of facelift flap and coverage with Saran Wrap

Topical NTG was started at the earliest sign of flap ischemia. The patients were instructed to apply NTG 2% ointment (E. Fougera & Co., Melville, NY) two times daily to the affected area(s) and cover the region with a small piece of Saran Wrap (Figure 1). 

The main outcome measure was the incidence of flap ischemia and skin slough in nicotine users who were placed on perioperative niacin and started topical NTG at the initial presentation of skin ischemia. The time needed to reverse flap ischemia and prevent skin slough was also recorded with the use of this therapy. 

Facelift surgery technique

A modified conservative technique was used in all patients who used nicotine products. The procedure is started with suction-assisted liposuction of the submental, submandibular areas. The neck skin is undermined with blunt and sharp dissection, taking care to stay superficial to the platysma. A “long flap” that involves complete undermining of the neck skin from the mastoid prominence to the contralateral mastoid prominence is not performed on any nicotine user. The postauricular flap is similarly elevated in a subcutaneous plane. The preauricular dissection is carried out in a radial fashion in the subcutaneous plane, superficial to the superficial musculoaponeurotic system (SMAS), for approximately 4-5 cm in non-nicotine users and 2-3 cm in smokers, extending anteriorly and inferiorly from the origin of the superior crus of the ear to the occipital incision. Dissection is limited to 2-3 cm in nicotine users to minimize flap undermining. After flap elevation is complete, liposuction of the jowl and neck areas is completed using a 4 mm blunt cannula. Next, imbrication of the SMAS is carried out with several interrupted 2.0 Vicryl sutures (Ethicon, Somerville, NJ). The skin is advanced in a superior posterior vector and closed without tension. The temporal incision is made back in the hairline, and dissection is performed in the subgaleal, supratemporalis fascia plane. Stainless steel staples are used in the hair-bearing areas. A 5.0 catgut suture (Ethicon, Somerville, NJ) is subsequently placed between each staple and used to close the pre- and postauricular wounds. All drains used during surgery are removed, and a circumferential pressure dressing is applied over the undermined areas.

## Results

The forty patients (age range 55-72 years [mean 63 years], all female patients) enrolled in the study consisted of 27 active nicotine users and 13 non-nicotine users with evidence of flap ischemia in the postoperative period. Most patients had other procedures performed simultaneously: blepharoplasty (37), rhinoplasty (4), pretrichial forehead lift (8), phenol chemical peel perioral (7), phenol chemical peel periorbital (3), dermabrasion perioral (4), dermabrasion nose (1), lip augmentation with SMAS (4), lip advancement (1). No patient had a simultaneous resurfacing procedure performed over the undermined facelift flap.

**Figure 2:**
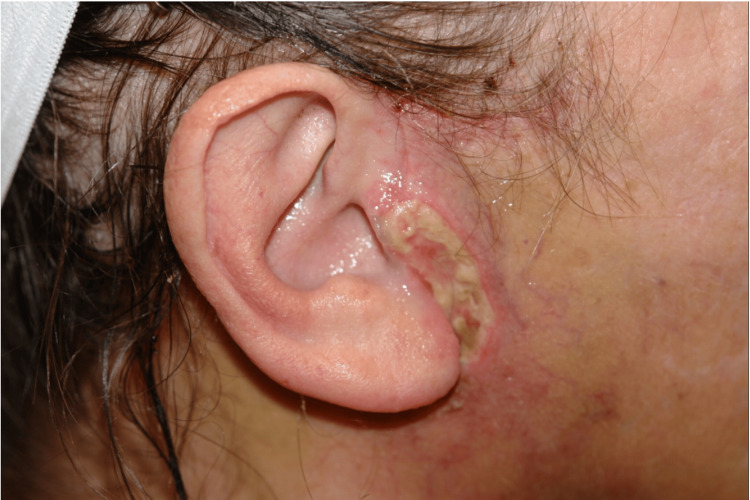
Full-thickness skin loss in a nicotine user

**Figure 3:**
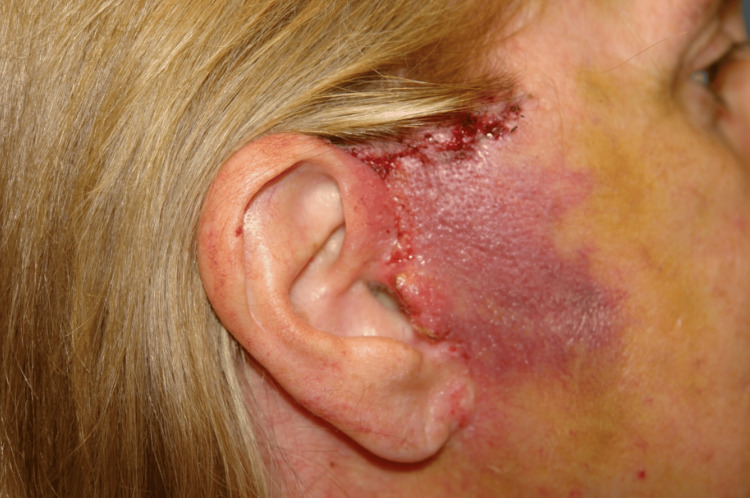
Preauricular ischemic skin in a nicotine user

**Figure 4:**
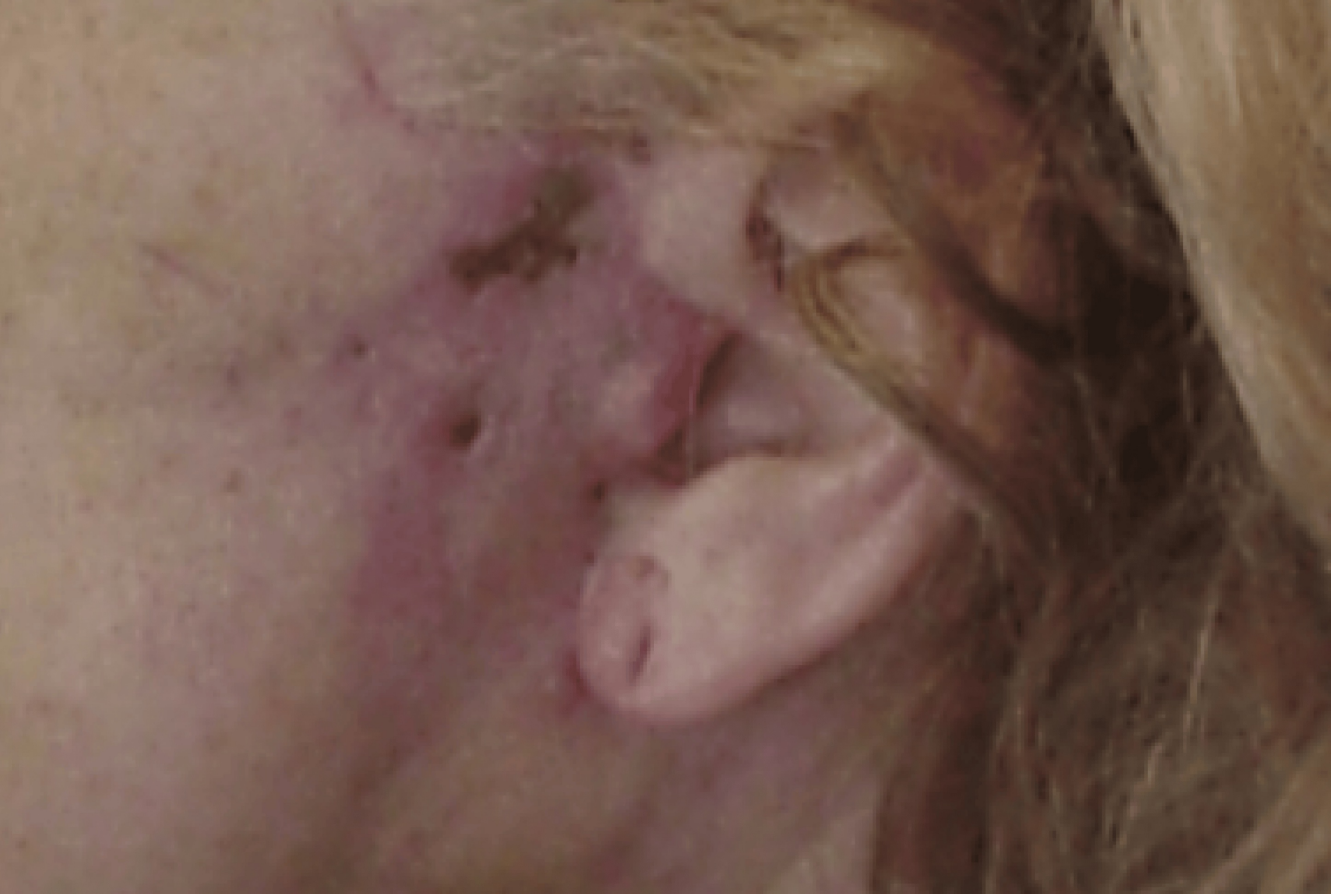
Improved appearance of ischemic skin flap three days after starting IR niacin and topical NTG IR, immediate release; NTG, nitroglycerin.

Flap ischemia was seen in five nicotine users (5/27 patients, 18.5%), and one of these patients had skin slough (Figure 2). Thirteen non-nicotine users had evidence of flap ischemia in the early postoperative period. Early signs of flap ischemia included venous congestion, poor capillary refill, and decreased temperature. Symptoms most often presented between postoperative days 1-3. Most cases of flap ischemia involved the immediate preauricular skin (12 patients) (Figure 3). Eight patients had ischemia of their postauricular flap only. Four patients had ischemia of both their pre- and postauricular flaps. As outlined above, all nicotine users continued perioperative niacin, and those with flap ischemia started topical NTG two times daily to the affected area(s). Non-nicotine users with flap ischemia were immediately started on IR niacin as outlined in Figure 1 and applied topical NTG. Flap ischemia improved in 3-5 days (mean 4 days) among the nicotine users. One patient who did have skin slough continued niacin, topical NTG, and local wound care, and her preauricular flap re-epithelialized in 18 days following skin necrosis. Among the 13 non-nicotine users with flap ischemia, seven patients had a “long” skin flap due to concurrent platysmaplasty being performed. Three patients had a tuck-up procedure several years following their primary rhytidectomy procedure. One patient had a facial hematoma in the early postoperative period. There was no incidence of skin slough in this patient population. Flap ischemia readily improved among these patients in 3-7 days (mean 5 days) after starting IR niacin and topical NTG (Figure 4). 

**Figure 5:**
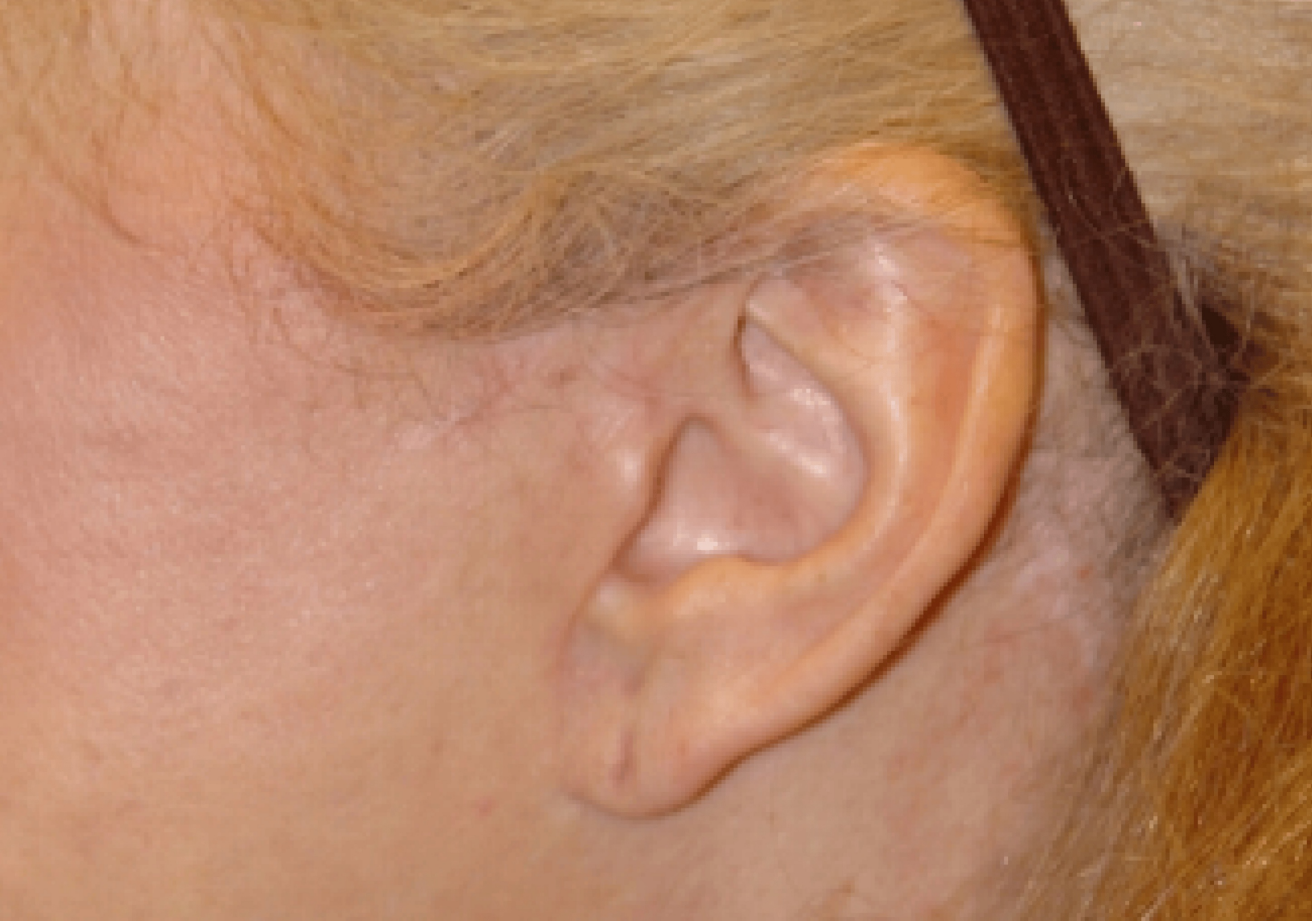
Healed preauricular skin three months after operation

There were no long-term complications. A 55-year smoker, who was taking a large dose of IR niacin (4 grams (g)/day), did experience transient tachycardia, chest palpitations, and hyperglycemia. She required a visit to the emergency room, and after a negative cardiac workup, was asked to stop her niacin. Overnight hospital admission was not required. There was one case of preauricular skin necrosis in a 58-year-old smoker who developed limited hypertrophic scarring after re-epithelialization. This responded well to serial cortisone injections without long-term sequelae (Figure 5). There were no cases of secondary infections in the study group.

## Discussion

Facelift surgery is one of the most popular cosmetic procedures performed in the head and neck region. Patient selection and preoperative counseling are critical to achieving favorable outcomes. Nicotine use is considered a relative contraindication to rhytidectomy due to the increased risk of flap ischemia and necrosis [1]. Nicotine amplifies norepinephrine (NE)-induced vasoconstriction and impairs endothelium-dependent vasorelaxation [9]. Additionally, cigarette smoking has long been associated with premature skin aging [10]. As more smokers pursue facial rejuvenation procedures, it becomes essential for surgeons to counsel these patients regarding perioperative risks, preventive measures, and realistic expectations. For example, in our practice, all nicotine users are informed during their preoperative consultation that due to their nicotine use, we can only expect to achieve 70-80% of the optimal surgical result. Ideally, patients would abstain from all nicotine products several weeks before and after surgery. However, our experience indicates that most patients continue nicotine use throughout the perioperative period. Despite this, we have found that rhytidectomy can still be performed safely and effectively in nicotine users when the perioperative protocol outlined in this article is followed.

Niacin (also known as nicotinic acid or vitamin B3) is a safe, water-soluble compound first discovered in 1873 [11]. It is widely used for treating hyperlipidemia and is known to raise high-density lipoprotein cholesterol (HDL-C) while lowering triglycerides and low-density lipoprotein cholesterol (LDL-C) [4]. The vasodilatory effect of niacin is mediated by prostaglandin D₂ and presents as sudden warmth, redness, itching, headache, or facial tingling [5,6]. These effects can counteract nicotine-induced vasoconstriction. While most published dosing recommendations for niacin are for lipid management (1-2 g/day for IR formulations, up to a maximum of 6 g/day) [12], the maximum for ER formulations is 2 g/day [13]. Side effects may include exacerbation of peptic ulcer disease (PUD), tachycardia, arrhythmias, hyperuricemia, transient hyperglycemia, and palpitations at high doses (>3 g/day) [14]. Recent data also support the safe use of niacin in diabetic patients with appropriate monitoring [15]. Contraindications include hypersensitivity to niacin, hepatic dysfunction, bleeding disorders, and active PUD [14]. As part of our preoperative clearance, all patients undergo a complete blood count (CBC), coagulation profile, electrocardiogram (EKG), and comprehensive metabolic panel (CMP), and niacin is avoided in patients with any contraindications.

Topical NTG is a potent vasodilator that promotes endothelial prostacyclin release and relaxation of vascular smooth muscle [8]. NTG is particularly useful for treating ischemic skin flaps because it can be applied directly to the affected area, preferentially dilating spastic vessels and improving localized perfusion [7,8].

In our experience, facelift surgery can be performed safely in nicotine users if appropriate precautions are taken. Patients are counseled thoroughly about the cardiopulmonary risks of continued nicotine use and are encouraged to abstain from nicotine both before and after surgery. Despite this guidance, all 27 nicotine users in our study continued using nicotine products during the perioperative period. The use of oral niacin and topical NTG has proven to be a valuable adjunct in managing these patients. Furthermore, this therapeutic approach has been effective in salvaging ischemic skin flaps even in non-nicotine users.

Our findings suggest that niacin and topical NTG may act synergistically to protect and salvage ischemic facelift flaps. Niacin’s flushing response provides visual evidence of perfusion, while topical NTG improves blood flow by dilating affected vessels. Nicotine also depletes endogenous niacin and displaces it from its receptor sites [16]; replenishing niacin in smokers may reduce nicotine’s adrenergic effects. In our practice, oral niacin combined with a conservative facelift technique prevented skin flap complications in 22 of 27 nicotine users. Among the five who experienced flap ischemia, four avoided progression to necrosis due to twice-daily administration of oral niacin and topical NTG. In the one case of full-thickness skin flap loss, the extent of necrosis was limited with this therapy.

Interestingly, early use of IR oral niacin and topical NTG also helped prevent necrosis in 13 non-smokers who developed flap ischemia. Risk factors in this group included aggressive flap dissection (e.g., during platysmaplasty), revision surgery, and hematoma formation. Prompt recognition and correction of underlying causes, along with this adjunctive therapy, helped avoid long-term complications.

The incidence of skin necrosis following rhytidectomy is reported to be between 1.1% and 3% [17]. Riefkohl et al. found a 19.5% incidence in active smokers compared to 5% in nonsmokers [18]. Among 247 facelifts performed at our clinic, only one case (0.4%) of skin slough occurred, which we attribute to the combined use of niacin, topical NTG, and conservative skin flap undermining. Among our 27 nicotine users, the incidence of flap necrosis was 3% (1/27).

This study aimed to present strategies to improve outcomes in nicotine users undergoing rhytidectomy. These include conservative flap dissection, patient education, perioperative abstinence from nicotine, oral niacin use, and early application of topical NTG. While nicotine cessation remains ideal, it is often difficult due to its addictive nature. Varenicline (Chantix®, Pfizer Inc., Mission, KS), a nicotinic acetylcholine receptor partial agonist, may help patients quit smoking by mimicking some of nicotine’s effects while also blocking receptor activation [19]. However, it should be used cautiously in patients with renal impairment and avoided in those with psychiatric disorders [18]. With coordination from the patient’s primary care physician, varenicline may provide a helpful adjunct to perioperative care.

Limitations

This study is limited by its retrospective design and relatively small sample size, especially within the nicotine-using subgroup. The absence of a randomized control group restricts the ability to draw definitive causal conclusions regarding the specific effects of niacin and topical NTG. Additionally, uniformity in surgical technique, timing of adjunctive treatment, and patient compliance could not be strictly controlled. Continued nicotine use in all cases also limits the ability to assess these therapies in abstinent patients. Finally, outcomes were based on clinical observation without objective measures such as perfusion imaging, which may introduce observer bias.

## Conclusions

As more patients seek facelift surgery, surgeons have an increasing need to counsel nicotine users on the risks associated with rhytidectomy in this patient population. Skin slough can be a particularly devastating complication among these patients. Our experience shows that surgery can be performed on nicotine users in a relatively safe manner by using a more conservative surgical technique, perioperative use of niacin, and topical NTG for early signs of flap ischemia. Skin necrosis can also be prevented in non-nicotine users who develop flap ischemia with early use of oral niacin and topical NTG. Hence, our findings may serve as a useful adjunct for surgeons advising patients on perioperative complications and their treatment following facelift surgery.
